# COVID-19 and patient-reported experience of general practice in England: an evaluation study

**DOI:** 10.3399/BJGPO.2024.0209

**Published:** 2025-12-19

**Authors:** Paul Allanson, Paul Logan

**Affiliations:** 1 School of Business, University of Dundee, Dundee, UK; 2 School of Economics, University of Edinburgh, Edinburgh, UK

**Keywords:** COVID-19, quality assurance, social sciences, statistics

## Abstract

**Background:**

The COVID-19 pandemic led to a rapid transformation of the operating model for GP practices in England, with a switch towards the use of remote rather than face-to-face appointments.

**Aim:**

To assess changes in the quality of general practice in England over the course of the COVID-19 pandemic based on patients’ views of their experiences.

**Design & setting:**

Analysis of practice-level multicategory response data on patient-reported experience measures (PREMs) from annual GP Patient Surveys from 2018–2023.

**Method:**

Healthcare quality changes (HQC) at both practice and national levels were assessed. An index sensitive to changes in the distribution of patient responses was used across the full set of PREM response categories, not just in the proportion meeting a binary quality threshold.

**Results:**

Patients’ reported experience of general practice improved nationally between the 2020 and 2021 surveys, in spite of the restrictions on the operation of GP practices. The reported experiences then fell sharply between 2021 and 2022 before resuming the pre-pandemic downward trend. Variation in HQCs at the practice level was considerable between all consecutive years.

**Conclusion:**

Changes in patients’ reports of their experiences of general practice over the course of the pandemic reflected broader shifts in public attitudes towards the NHS as well as real changes in the nature and quality of service delivery.

## How this fits in

This article makes use of a recently proposed framework for the comparative evaluation of healthcare quality profiles. It provides a more detailed picture of changes in general practice quality in England over the course of the COVID-19 pandemic than is provided by routine publications that report the change in the proportion of patients describing their overall experience as good. The results strongly suggest that the use of patient-reported experience measures (PREMs) to monitor national trends in general practice quality was confounded by pronounced shifts in patients’ reporting behaviour over the course of the pandemic. The results also indicate that the choice of a face-to-face appointment is likely to be a higher priority for patients than the right to digital-first primary care when it comes to the post-pandemic provision of general practice.

## Introduction

The COVID-19 pandemic led to GP practices in England being advised on 19 March 2020 to triage appointments by telephone or online and to undertake all care that could be done remotely via appropriate channels.^1^ Telephone and video or online consultations made up just 15.2% of appointments in February 2020 but rose sharply to 50.4% at the height of the first pandemic wave in April 2020 before falling back gradually to 42.6% by April 2021 and 28.2% by April 2023.^2^ This paper assesses the impact of the pandemic on patients’ views of their experience of general practice in England using data from the GP Patient Survey (GPPS), which is sent out annually to over 2 million adult patients registered at a GP practice. Understanding patients’ attitudes to the switch in consultation mode is of particular importance given the debate as to whether the change should be reversed following the end of the pandemic.^3–6^ More generally, the analysis provides insight into public attitudes towards the NHS at a time of unprecedented stress on the healthcare system.

Patient experience is an intrinsically important dimension of healthcare quality.^7^ The GPPS gives patients the opportunity to feed back about their GP practice and other local NHS services, with the 2020 survey providing a snapshot of patient experience in the immediate run-up to the pandemic, given that the fieldwork was almost all complete before the first UK national lockdown on 23 March 2020. Our main analysis uses data on the proportions of patients in each GP practice who describe their overall experience of the practice as very poor, fairly poor, neither good nor poor, fairly good, and very good. According to the 2021 GPPS national report, ‘*more patients described their* [overall] *experience as good* [i.e. fairly or very good] *than in 2020, in contrast with the previous downward trend’*.^8^ Inspection of the data further reveals that the distribution of responses became more polarised in 2021, with higher proportions of both very good and very poor responses. This paper explores national changes in general practice quality using an index that is sensitive to changes in the proportions of patients in all response categories, not just the change in the proportion describing their experience as good, as well as presenting evidence on the variation in quality changes at the practice level.

## Method

### Measurement of healthcare quality change

Regularly conducted surveys of patient experience in a range of primary and secondary care settings in England^9^ provide a valuable resource for monitoring national trends in quality of care.^10^ Patients are typically asked to rate their care experience by choosing between one of several ranked categories, yielding polytomous categorical response measures which, owing to their ordinal or qualitative nature, lack a well-defined mean. To overcome this limitation, changes in patient-reported experience measures (PREMs) are commonly reported in terms of changes in the proportion of patients meeting a binary quality threshold as in the 2021 GPPS,^8^ but dichotomisation results in a loss of information with the measured quality change dependent on the choice of threshold. This paper employs an alternative approach that is based on the assessment framework proposed in Allanson *et al* ^11^ for the comparative evaluation of healthcare quality profiles, and makes full use of the ordinal information embodied in multicategory response data.

Specifically, the change in healthcare quality between two dates is defined as the difference in the chances that the experience of a randomly chosen patient at the end date was better rather than worse than that of a similarly chosen patient at the start date. By way of illustration, consider an example in which the proportion of patients reporting their care experience as either ‘poor’, ‘OK’, or ‘good’ is (0.5, 0.1, 0.4) and (0.4, 0.3, 0.3), respectively, at the start and end dates. Hence the probabilities that a randomly chosen patient at the end date reports better, the same, or worse care than a similarly chosen patient at the start date will be (0.5*[0.3+0.3]) + (0.1*0.3) = 0.33, (0.5*0.4) + (0.1*0.3) + (0.4*0.3) = 0.35 and (0.1*0.4) + (0.4*[0.4*+0.3]) = 0.32 respectively, with the healthcare quality change (HQC) index equal to the difference 0.33 − 0.32 = 0.01. HQC will take a value of zero if the quality profiles at the two dates are equivalent, although this does not necessarily imply that they are identical; a maximum value of one when the worst quality of care experienced at the end date is strictly better than the best quality experienced at the start date; and a minimum value of minus one when the opposite is the case. For binary response measures, HQC is simply the change in the proportion reporting ‘good’ (rather than ‘not good’) care.

### Data

Data were drawn from the GPPS, which is described in detail in the 2023 GPPS technical annex.^12^ In brief, it is a postal survey of adult GP registered patients, which has been running annually since 2017 with a single fieldwork period from January to early April. The survey includes all GP practices with eligible patients in England, with the random selection of patients in each practice based on a proportionately stratified, unclustered design. Since 2022, the sample size for each practice has been determined to deliver at least 100 responses, having previously been chosen to ensure confidence intervals were as consistent as possible across practices. Analysis has shown that responses received in the last 2 weeks of fieldwork in 2020, following the national lockdown, were too small a proportion of the total to affect the overall results for the survey as a whole, even though they were generally more positive compared with the same period in 2019.^13^

The survey includes questions about a range of topics including patients’ local GP and other NHS services, their experience of making a general practice appointment and their last such appointment, and about the patients themselves.^14^ Our main analysis is based on responses to the question: 'Overall, how would you describe your experience of your GP practice?' This question was introduced in 2011 following cognitive testing,^15^ with overall experience identified as a key indicator of practice quality in a subsequent online feedback exercise.^16^ The questionnaire was considerably redeveloped in 2018, limiting comparability with results from earlier years even where question wording remained similar owing to changes in context,^16^ and again in 2024.^17^ More minor changes were also made in 2021, 2022, and 2023 to reflect changes in the delivery of primary care services over the course of the COVID-19 pandemic,^18^ with trends only reported for those questions not deemed subject to significant change. Accordingly, we restrict our analysis to responses to this set of questions between 2018 and 2023. Table 1 shows that more than 700 000 completed questionnaires were returned in each of these years, with the national response rate varying between 28.6% and 35.1%.

**Table 1.** Sample characteristics

	2018	2019	2020	2021	2022	2023
Total completed GPPS questionnaires	850 206	770 512	739 637	770 512	719 137	759 149
*National response rate (%*)	*34.1*	*33.1*	*31.7*	*35.1*	*29.1*	*28.6*
All general practices in GPPS	7254	6999	6821	6658	6507	6418
Cross-sectional sample size	7185	6926	6734	6591	6462	6401
Balanced panel: consecutive pairs of years	~	6923	6728	6584	6458	6372
2018 year	~	6923	6726	6579	6448	6362

Source: own calculations from the GPPS data. GPPS = GP Patient Survey

GPPS data are published as official statistics in the form of a searchable online database^19^ to help inform patients’ choices. Our main analysis is based on all practices for which response data were available in any given year, which is slightly less than the total number of surveyed practices owing to the suppression of data to protect the confidentiality of individuals (see Table 1). Specifically, we made use of the weighted practice results that more accurately reflects the patient population in each practice by correcting for the sampling design and to reduce the impact of non-response bias. We also made use of the online GPPS analysis tool in supplementary analyses to generate national cross-tabulations of weighted responses to the 2021 survey.

### Quantitative analysis

HQC indices were calculated using the approach employed in the numerical example, with associated standard errors generated via Monte Carlo simulation of the data-generating process. Specifically, the GPPS provides for each practice an estimate of the true proportion of patients in each response category in any given year based on a random sample drawn separately for that year. Hence, simulated data on practice-level quality profiles for each year were generated as independent random draws from a multinomial distribution with parameters set equal to the number of survey responses and response category proportions for each practice. Aggregation of the simulated practice profiles to the national level employed practice weights based on the number of registered patients aged ≥16 years in the December before each survey.^20^ All analysis was conducted using Stata (version 15.1).

## Results

Table 2 reports our headline finding that the patient-reported quality of general practice improved nationally between 2020 and 2021, with this improvement more than offsetting the deterioration that occurred over the preceding 2 years. In particular, there was a 4.47 percentage point (pp) higher chance that a patient chosen at random from anywhere in England in 2021 would have reported a better rather than worse overall experience than one similarly chosen in 2020. In contrast, the 1.21 pp increase in the proportion reporting a good experience in 2021 only offset the fall observed in the previous year, largely because this measure fails to capture the marked increase in the proportion of patients reporting their experience as not just fairly but very good in 2021. Both measures indicate a substantial fall in general practice quality between 2021 and 2022, with the further decline in 2023 more in line with the pre-pandemic trend.

**Table 2.** Overall experience of GP practice, England 2018–2023

		2018	2019	2020	2021	2022	2023
National overall experience profiles			Percentages				
Very good	} Good	46.20	45.17	43.57	48.24	37.67	36.79
Fairly good		37.56	37.76	38.20	34.73	34.71	34.53
Neither good nor bad		10.21	10.60	11.22	10.36	14.00	14.53
Fairly bad		4.15	4.42	4.74	4.29	8.06	8.36
Very bad		1.88	2.06	2.28	2.37	5.56	5.79
National healthcare quality change (HQC) indices			Percentage points				
HQC from previous year		~	–1.34	–1.99	4.47	–14.78	–1.30
			0.10	0.10	0.10	0.09	0.08
HQC from 2018		~	–1.34	–3.32	1.21	–13.89	–15.22
			0.10	0.09	0.12	0.10	0.10
Change in ‘Good’ percentage from previous year		~	–0.83	–1.16	1.21	–10.59	–1.06
			0.06	0.07	0.08	0.07	0.09
Change in ‘Good’ percentage from 2018		~	–0.83	–1.99	–0.79	–11.38	–12.44
			0.06	0.06	0.07	0.08	0.08

Source: own calculations from GPPS data. Monte Carlo simulated standard errors based on 50 trials are in italics. GPPS = GP Patient Survey. HQC = healthcare quality change

Further analysis suggests that patients’ responses would likely have been even more positive in 2021 if it had not been for the switch in consultation mode given that patients seen at their own GP practice were more positive about all specific aspects of their last appointment compared with those only spoken to on the phone or online (see Supplementary Table S1). Moreover, patient subgroups that might have been expected to place a higher value on the convenience of remote consultations — such as full-time paid workers and those who had avoided making an appointment in the preceding 12 months owing to lack of time — show similar if less strong preferences for being seen at their own GP practice. Nevertheless, patients’ support for local NHS services in 2021 was not uncritical, with much greater increases in the rating of some aspects of their GP practice, most notably the availability of appointment times, than of others (see Supplementary Table S2). Nor was it unconditional, with Supplementary Table S2 also reporting a marked deterioration in patients’ experiences of local NHS dentist services, which were more severely constrained by COVID-19 restrictions.

**Figure 1.**
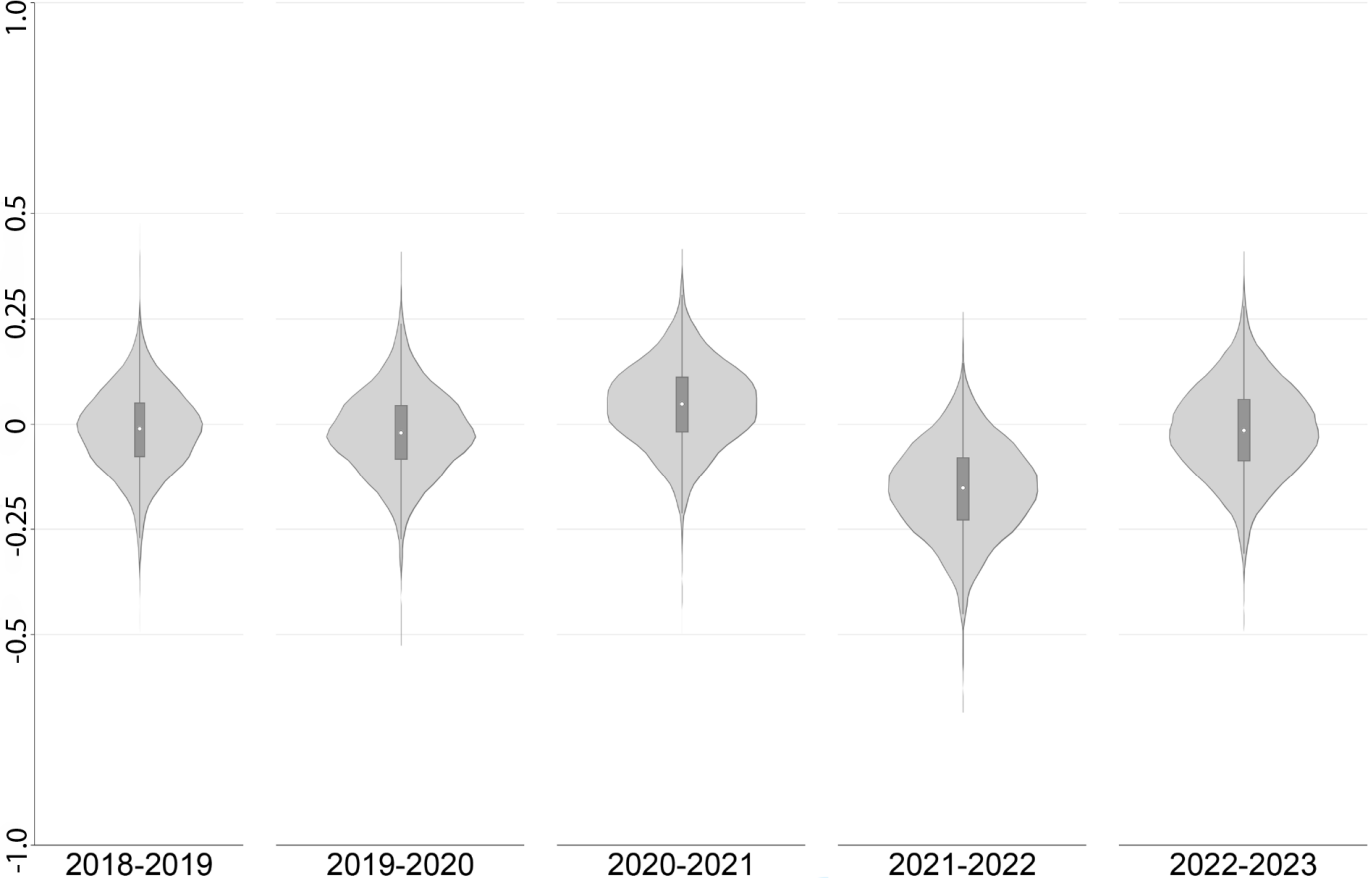
Violin plots of annual GP practice-level changes in healthcare quality. Source: own calculations from GPPS data. GPPS = GP Patient Survey

The national HQC results are largely driven by changes in practice-level quality profiles given the low turnover of practices between years. Figure 1 illustrates the considerable variation in practice-level HQCs, with the most improved practice in 2021 recording a positive 40.08 pp difference in the chances of a better rather than worse overall experience compared with a negative 48.23 pp difference for the worst performer. Healthcare quality is estimated to have fallen between 2020 and 2021 at just over one-third of practices despite the national improvement in general practice quality. Nevertheless, there was no appreciable change in the degree of variation in quality across practices: the absolute difference in the chances that a patient from a typical practice would have reported a better rather than worse overall experience than one from anywhere in England was 15.84 pp in 2021, compared with an average absolute difference of 17.24 pp across all 6 years. Indeed, the spread of the GP practice comparative quality distributions were broadly similar in all 6 years (see Supplementary Figure S1).

## Discussion

### Summary

The paper uses GPPS data to generate fresh insights into the impact of the COVID-19 pandemic on the quality of general practice in England. Patients rated the overall experience of their GP practice more highly in 2021 than in 2020 despite pandemic restrictions on the operation of general practices, most notably the prescribed switch towards the use of remote rather than face-to-face appointments.

### Strengths and limitations

The main strength of the study is that it is based on information from the GPPS, which offers a large-scale, annual survey of all GP practices with eligible patients in England. The HQC index makes full use of the practice-level multicategory response data made publicly available from the GPPS on the quality of health care received by patients. Nevertheless, it should be borne in mind that the GPPS is a repeated cross-sectional survey and therefore does not track the healthcare experience of individual patients.

### Comparison with existing literature

A credible explanation of at least part of patients’ more positive rating of their local GP services in GPPS 2021 was that a more supportive attitude towards the NHS in the pandemic led to a change in reporting behaviour. Evidence of such public goodwill can be adduced from both the ‘Clap For Our Carers’ campaign during the first national lockdown in Spring 2020^21^ and the findings of an online survey conducted in late November 2020.^22^ Moreover, the waning extent of any resultant ‘gratitude bias’^23^ likely accounts for a large part of the substantial drop in patients’ overall rating of their GP practice between GPPS 2021 and 2022 as public expectations of the NHS began returning to normal,^24^ with tracking data reported in Buzelli *et al*,^25^ indicating an exceptionally sharp deterioration between March and November 2021 in public perceptions of NHS standards over the preceding 12 months.

An October 2021 YouGov poll found that 66% of all adults would prefer a face-to-face appointment compared with 3% for a video-call, with 25% not minding what type of appointment they were offered.^26^ Indeed, making it easier to get face-to-face GP appointments has been consistently ranked as a top NHS priority by just over one-quarter of responders to a biannual, online survey first conducted in November 2021.^27^ Recent research^28^ using GPPS data concludes that online consultation systems have the potential to improve patients’ overall experience but will only do so if careful consideration is given to design and implemenation issues.

### Implications for research and practice

The findings suggest that changes in patient-reported general practice quality over the course of the pandemic reflected broader shifts in public attitudes towards the NHS as well as objective changes in service delivery. The choice of a face-to-face appointment is likely to be of more importance to patients than the right to digital-first primary care envisaged pre-pandemic in the 2019 NHS Long Term Plan^29^ when it comes to deciding on the post-pandemic balance between consultation modes: current NHS England plans commit to *‘respecting* [patients’] *preference for a call, face-to-face appointment, or online message’* ^30^ while providing additional funding for ‘*higher-quality digital tools that enable the shift to online*’.^30^ Further work on the impact of the pandemic on healthcare quality would benefit from the use of longitudinal data to analyse changes in individual patient experience.

## Data

The data that support the findings of this study were derived from the following resources available in the public domain: NHS Digital: Patients registered at a GP Practice (various months). Available at: https://digital.nhs.uk/data-and-information/publications/statistical/patients-registered-at-a-gp-practice#past-publications. NHS England: The GP Patient Survey (various years). Available at: https://gp-patient.co.uk/surveysandreports.
